# Effect of simulated microgravity conditions of hindlimb unloading on mice hematopoietic and mesenchymal stromal cells

**DOI:** 10.1002/cbin.11432

**Published:** 2020-08-08

**Authors:** Shiyun Dai, Fanxuan Kong, Chao Liu, Fengjun Xiao, Xiwen Dong, Yikun Zhang, Hua Wang

**Affiliations:** ^1^ Graduate School Anhui Medical University Hefei Anhui China; ^2^ Department of Experimental Hematology Beijing Institute of Radiation Medicine Beijing China; ^3^ Binzhou Medical University Yantai Shandong China; ^4^ Beijing Key Laboratory for Radiobiology Beijing Institute of Radiation Medicine Beijing China; ^5^ Department of Hematology PLA Strategic Support Force Characteristic Medical Center Beijing China

**Keywords:** hematopoietic cells, hindlimb unloading, mesenchymal stromal cells, microgravity, morphology, phenotype

## Abstract

Conditions in space, such as microgravity, may affect the hematopoietic and bone marrow‐derived mesenchymal stromal cells (BM‐MSCs) of astronauts. However, to date, few detailed phenotype change data about the different type of hematopoietic cells have reported. In this study, C57BL/6 mice were randomly divided into two groups: a control group (control) and a hindlimb suspension group (treated). After four weeks of hindlimb suspension, we found that this simulated microgravity (sµg) condition could increase the percentage of monocytes and macrophages and decrease the percentage of B lymphocytes and mature red cells in bone marrow. The percentage of B lymphocytes in the spleen and the red blood cell count in peripheral blood also decreased, consistent with the response of bone marrow. The cytoskeleton in the BM‐MSCs was disrupted. The expression levels of hematopoietic‐related genes, such as fms‐like tyrosine kinase‐3 ligand, granulocyte‐macrophage colony stimulating factor, interleukin‐3, and adipogenic differentiation associated genes, leptin and proliferator‐activated receptor γ type 2, were upregulated under sµg conditions. These results indicated that simulating microgravity can affect the phenotype of certain types of hematopoietic cells and the morphology and gene expression pattern of BM‐MSCs.

**Abbreviations**
BM‐MSC: bone marrow‐derived mesenchymal stromal cell
CFU: colony‐forming unit
COL‐1: collagen‐I
Flt‐3L: fms‐like tyrosine kinase‐3 ligand
GM‐CSF: granulocyte‐macrophage colony stimulating factor
HSC: hematopoietic stem cell
HU: hindlimb unloading
IL‐3: interleukin‐3
LSK: Lin‐Sca‐1^+^c‐kit^+^
OC: osteocalcin
OS: osteonectin
PBS: phosphate buffered saline
PPARγ2: proliferator‐activated receptor γ type 2
RBC: red blood cell
RUNX2: RUNT‐related transcription factor 2
RWV: rotating wall vessel
SCF: stem cell factor
SEM: standard error of the mean
WBC: white blood cell

## 1 INTRODUCTION

The environment in space includes exposure to various forces, including microgravity, radiation, alterations in circadian rhythm, and extreme temperatures. Studies of humans and animals that have experienced space flight (SF) have shown that many tissues and organ systems display some measurable changes. In some cases, these changes are minor, and their relevance to astronaut health has not been determined (Globus and Morey‐Holton, 2016). Microgravity is known to enhance cancer risk and influence biological systems, including bone, muscle, heart, and brain (Ulbrich et al., 2014). Human SF missions have resulted in some hematological anomalies. These effects of microgravity on hematological function have drawn the attention of researchers to those effects on the health of astronauts from the outer space.

Many studies have shown that microgravity dramatically affects cell morphology, proliferation, differentiation, and signal transduction. Currently, the influence of microgravity on isolated cells is being studied in vitro (Y. N. Zhang et al., 2018). However, the results obtained from in vitro experiments do not represent the in vivo situations. Considering that it is not practicable to gather enough tissue samples from astronauts for a thorough investigation, hindlimb suspension has been developed as a means to simulate the effects of microgravity, enabling studies of the effects of microgravity on the biology and behavior of mice (Ulbrich et al., 2014).

Bone marrow mainly includes two types of cells with respect to their origin—hematopoietic and mesenchymal (Ozcivici, 2013). The morphology, phenotypes, and differentiation of these cells may all be affected by microgravity. Davis’ group investigated the in vitro effects of SF on CD34^+^ hematopoietic cell proliferation and differentiation during the space shuttle missions STS‐63 (Discovery) and STS‐69 (Endeavour). (Blaber, Sato, & Almeida, 2014; Davis et al., 1996). Domaratskaya, Michurina, et al. (2002) analyzed the effect of SF on the clonogenic hemopoietic cells numbers of newts. Sotnezova, Markina, Andreeva, and Buravkova (2017) evaluated the content of myeloid stem colony‐forming unit (CFU) in mice bone marrow karyocytes after a 30‐day Bion‐M1 pace flight and observed a significant decrease in the number of erythroid progenitors, including common myeloid precursor after the SF. Markina, Andreeva, Andrianova, Sotnezova, and Buravkova (2018) also evaluated the effects of 30‐day SF on biosatellite on the mononuclear cells (MNCs) of murine bone marrow progenitors and discovered the total hematopoietic CFU number decreased. Huang et al. (2009) studied the differentiation of rat bone marrow mesenchymal stem cells in vitro under simulated microgravity (sµg). Markina, Andrianova, Shtemberg, and Buravkova (2018) studied the effect of 30‐day hindlimb unloading (HU) on the clonogenic and differentiation potential of bone marrow stromal progenitors in mice. They found clonogenic and differentiation activity of stromal cells decreased after unloading. Above mentioned results from real and sµg, long or short time, including rodent experiments, mainly focused on the cell count, function and morphology. Thus, detailed phenotype data describing these changes also need further study.

To observe whether sµg, which specifically affects the muscular and skeletal systems in rodents, can also affect other systems, such as the hematopoietic system, and in what degree they will be affected, hindlimb suspension was used in this study to evaluate the effects of microgravity simulation on hematopoietic and mesenchymal stromal cells (MSCs) in vivo.

## 2 MATERIALS AND METHODS

### 2.1 Hindlimb suspension

6–8‐week‐old C57BL/6N mice, with body weights of 20–22 g, were purchased from Vitalriver Corporation (Beijing, China) and acclimatized for 1 week in an air‐conditioned room at a temperature of 23±2°C with 12 h/12 hr light‐dark illumination cycles and humidity at 45–50%. The mice were randomly divided into a control group (control, *n* = 7) and a hindlimb suspension group (treated, *n* = 14). Treated mice were subjected to hindlimb suspension according to the method of Morey‐Holton and Globus (1998) and Y. N. Zhang et al. (2018). Briefly, hindlimbs were suspended at a 30° angle using a paper clip, adhesive tape, and a metal tail harness. The tail harness was attached to a swivel buckle mounted on a guide wire running the length of the cage via a metal chain. Using this setup, mice were able to access all areas of the cage. The hindlimb suspension was maintained for four weeks. All animal experiments were approved by the Ethics Committee of the Beijing Institute of Radiation Medicine (IACUC‐AMMS‐13‐2017‐024), and all procedures were conducted in accordance with relevant guidelines and regulations.

### 2.2 Peripheral blood cell counts

Twenty microliters of heparin anti‐coagulated peripheral blood samples were collected at Days 7, 14, 21, and 28 of HU from venae angularis. white blood cell (WBC), platelet (PLT), and red blood cell (RBC) counts were determined using a hematology analyzer CA500 (LanQiao Medical Technology Co., Ltd., Shan Dong, China).

### 2.3 Analysis of the surface markers of hematopoietic cells by flow cytometry

Femoral bones were sampled, the bone marrow was flushed out by drawing and expelling with a syringe and single cells were collected. Spleens were collected and grounded to obtain a single splenocyte. Heparin anti‐coagulated peripheral blood samples were collected from each group. The splenocyte and peripheral blood cells were lysed by 1×RBC lysis buffer (Beyotime, Shanghai, China) for 10 min. Surface markers for each cell type were quantified by flow cytometry and the cell number used for analysis was 1×10^6^/tube.

Monoclonal antibodies APC‐Cy7‐Lin^−^, PE‐c‐kit, and PE‐Cy7‐sca‐1 were used for the detection of LSK cells in bone marrow. Monoclonal antibodies against APC‐B220, PerCp‐CY5.5‐Ly6C, APC‐Ly6G, and DAPI‐F4/80 were used for the detection of granulocytes in bone marrow. Monoclonal antibodies PE‐CD71 and APC‐Ter119 were used for the detection of RBCs in bone marrow. Monoclonal antibodies FITC‐CD3, PerCp‐CY5.5‐CD4, PE‐CD8, and APC‐B220 were used to detect the phenotype of lymphocytes in the spleen. Monoclonal antibodies against APC‐CD3, PerCp‐CY5.5‐CD4, and PE‐CD8 were used to detect the phenotype of T lymphocytes in peripheral blood. Monoclonal antibodies FITC‐CD4, APC‐CD25, and PE‐FoxP3 were used to detect the phenotype of Tregs in peripheral blood. For each count, 1×10^6^ cells were washed twice with phosphate buffered saline (PBS), resuspended in 50 μl PBS containing monoclonal antibodies, and incubated for 30 min at 4°C. Cells were then washed twice and resuspended in 500 μl PBS. A flow cytometer (FACSCalibur, BD Biosciences) was used for fluorescence analysis. Percentages of positive cells were evaluated based on fluorescence intensity.

### 2.4 Hematopoietic progenitor cell colony‐forming cell (CFC) assays

Bone marrow cells were dispersed into PBS with 10% fetal bovine serum (FBS; HyClone, Logan, UT) and 1% penicillin and streptomycin (Gibco, Grand Island, NY). Viable bone marrow cells were quantified using a cell counter (EVE™, NanoEnTek, Korea) to exclude apoptotic and dead cells. CFC assays were performed to evaluate functional hematopoietic stem and progenitor cell content. Whole bone marrow cells were plated onto Methylcellulose‐based medium with recombinant cytokines for mouse cells, MethoCult™ GF M3434 (StemCell Technologies, Vancouver, BC, Canada). Colonies were scored on Day 10.

### 2.5 Fluorescence immunocytochemistry

Mouse bone marrow‐derived MSCs (BM‐MSCs) adherent culture was developed by seeding 1×10^6^ bone marrow mononuclear cells in α‐minimum essential medium (α‐MEM; Gibco, NY) containing 10% FBS (HyClone, Logan, UT) in 12‐well plate. About 12 days after culture, mouse BM‐MSCs were harvested and labeled with FITC‐CD31, FITC‐CD34,PE‐CD29, and PE‐Sca‐1 and detected by using flow cytometry. At the same time, 1,000 cells were cultured in the dishes and their cytoskeleton were visualized by using confocal microscope. Briefly, cells were fixed with 4% paraformaldehyde for 20 min, washed three times for 5 min/wash in PBS, and incubated in 0.01% Triton X‐100 at room temperature for 30 min. Cells were then blocked for 1 hr with 1% normal goat serum in blocking solution and incubated with a mouse antibody against rhodamine‐phalloidin (1:200; Sigma) for 2 hr to detect microfilaments. Cells were washed three times with PBS. After washing, cell nuclei were counterstained with diamidinophenylindole for 5 min. Cells were viewed and photographed by using a confocal microscope (Ultra view VOX, PerkinElmer).

### 2.6 Expression of hematopoietic promotion‐ and differentiation‐related genes in mouse cultured BM‐MSCs

Mice were sacrificed on Day 28 of microgravity simulation, and bone marrow were rinsed from femoral bone and cultured in α‐MEM medium containing 10% FBS for about 12 days. To detect changes in the expression of hematopoietic promotion‐ and differentiation‐related genes, cultured BM‐MSCs total RNA was isolated with TRIzol reagent (Life Technologies, Carlsbad, CA). cDNA was quantified by real‐time quantitative polymerase chain reaction (RT‐qPCR) using β‐actin for normalization (Table 1). We used 2^−ΔCt^ to represent the expression level. RT‐qPCR was conducted in ABI 7500 Fast PCR System (Applied Biosystems, CA).

**Table 1:** The primer sequence for qRT‐PCR

Gene	Accession number	Direction	Sequence (5′–3′)	Product length
mActin	NM_007393.5	Forward	AGG CCA ACC GTG AAA AGA TG	186
		Reverse	TGG CGT GAG GGA GAG CAT AG	
mRunx2	NM_001145920.2	Forward	CCA GTA TGA GAG TAG GTG TC	107
		Reverse	CTG CCT GGG ATC TGT AAT CT	
mOC	NM_007541.3	Forward	GCA GGA GGG CAA TAA GGT AG	147
		Reverse	GCG GTC TTC AAG CCA TAC TG	
mLeptin	NM_008493.3	Forward	ATA GCC AAT GAC CTG GAG AAT C	196
		Reverse	CCA ACT GTT GAA GAA TGT CCT G	
mPPAR‐γ2	NM_001127330.2	Forward	CCA AGA ATA CCA AAG TGC GAT C	174
		Reverse	TCA CAA GCA TGA ACT CCA TAG T	
mFlt‐3L	NM_013520.3	Forward	GGC CGT CAA TCT TCA GGA CG	95
		Reverse	ACC CTG CCA CAG TCT TCA GT	
mGM‐CSF	NM_009969.4	Forward	AGG GAC CAA GAG ATG TGG CA	82
		Reverse	CTT CCG CTG TCC AAG CTG AG	
mIL‐3R	NM_008369.1	Forward	TTA CCC TCG GAA GCT CAG CA	104
		Reverse	GCA CCG TAG CCA CTG AAG TC	
mSCF	NM_001347156.1	Forward	GCC AGC TCC CTT AGG AAT GA	108
		Reverse	CGA AAT GAG AGC CGG CAA TG	

### 2.7 Statistics

Data were analyzed using one‐way analysis of variance statistical analysis. The data are expressed as the mean ± *SEM* (standard error of the mean). GraphPad Prism version 6 (GraphPad Software) was used for statistical analysis. Student's t‐test was used to evaluate the statistical significance. In all of the analyses, *p* < .05 was considered to be significant.

## 3 RESULTS AND DISCUSSION

The rodent HU model is thought to mimic microgravity conditions and has been applied to characterize the effects of SF on integrated physiological, organ‐specific, and mechanistic responses (Globus and Morey‐Holton, 2016). In this study, we used an HU model (Figure S1) to observe the effects of sµg on hematopoietic cells and BM‐MSCs and found that some characteristics and/or morphology of these cells were changed.

### 3.1 Simulated microgravity decreased RBC counts in peripheral blood

**Figure 1:**
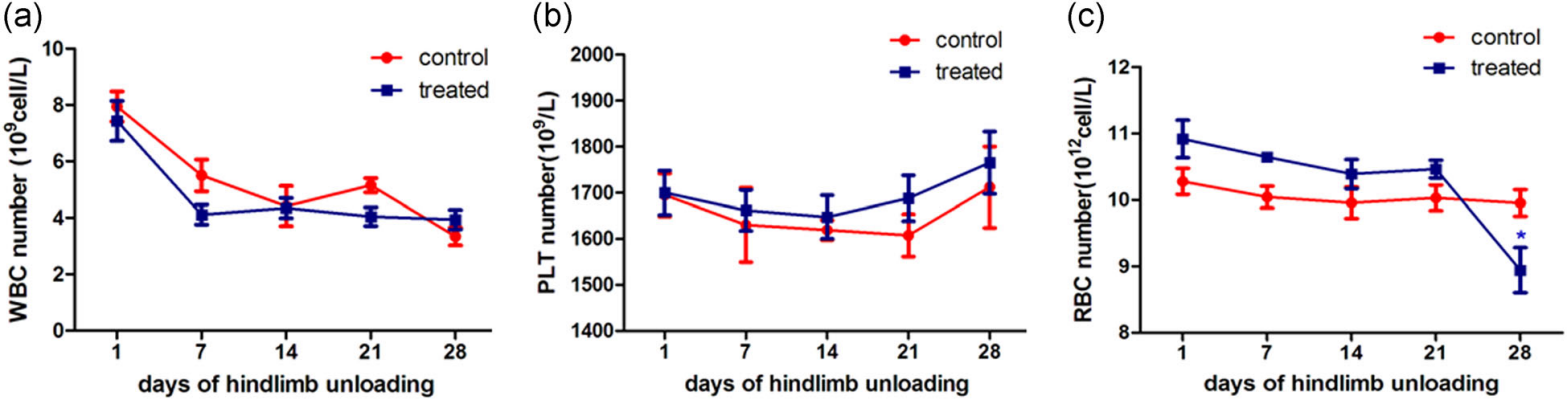
Blood cell counts in peripheral blood at different time points. (a) White blood cell number. (b) Platelet number. (c) Red blood cell number

To evaluate the effect of sµg on blood cell count in our study, mice underwent hindlimb suspension, and peripheral blood samples were taken at different time points. We did not observe significant changes in peripheral blood WBC (Figure 1a) and PLT (Figure 1b) counts after sµg. We only detected decreased RBC numbers (Figure 1c) in peripheral blood at Day 28 of microgravity simulation.

These results are not totally consistent with earlier reports that both human crew members and animals have shown changes in peripheral blood leukocytes (Chapes, Mastro, Sonnenfeld, & Berry, 1993; Gridley et al., 2009; Stowe, Sams, & Pierson, 2011).

### 3.2 The phenotype change of bone marrow hematopoietic cells under simulated microgravity

Some hematological anomalies have been found after human SF missions. Therefore, an increasing number of researchers are devoting attention to the effects of microgravity on hematological functions relevant to astronauts’ health. Most experiments to date have used isolated hematopoietic cells. Though techniques for the identification of hematopoietic stem cells (HSCs) have been developed recent years, actual detailed SF and simulated weightlessness data on HSCs still limited compared with data for other cell types.

**Figure 2:**
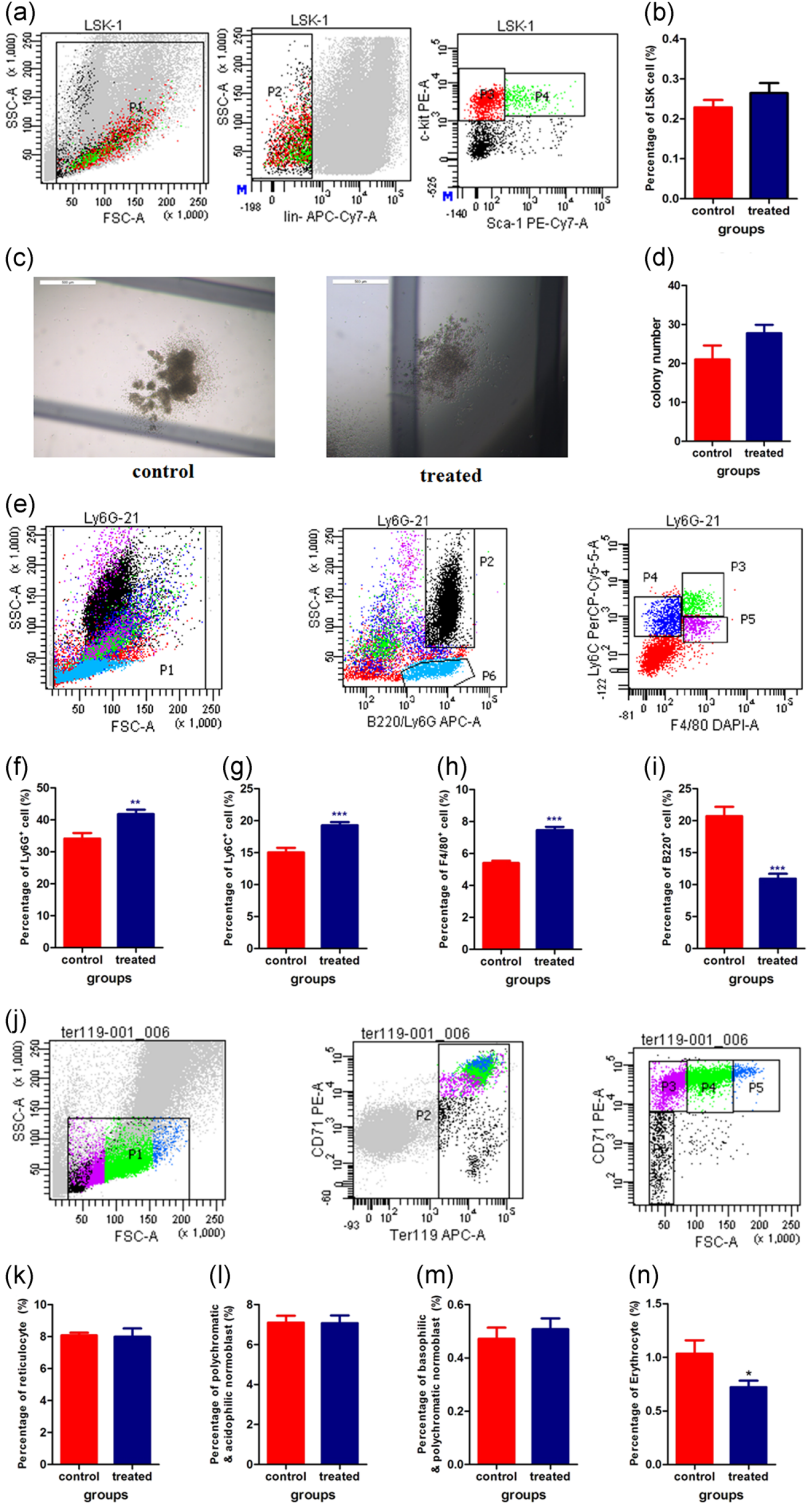
Effects of simulated microgravity on bone marrow hematopoietic cells. Bone marrow cells were collected and flow cytometry was used to detect phenotypes of bone marrow hematopoietic cells (a,b, e–n), and colony‐forming unit (CFU) assays were performed to measure functional hematopoietic stem and progenitor cell content (c,d). (a) Representative flow cytometric analysis of Lin‐Sca‐1^+^c‐kit^+^ (LSK). P4 represents Lin^‐^C‐kit^+^SCa‐1^+^ hematopoietic stem cells. (b) Quantification of the percentage of LSK cells. (c) Representative colony of bone marrow cells. (d) Statistical analyses of colony numbers. (e) Representative flow cytometric analysis of macrophages, granular leukocytes, monocytes, and B lymphocytes. (f–i) The percentages of Ly6G^+^ (f), Ly6C^+^ (g), F4/80^+^ (h), and B220^+^ (i) cells in bone marrow. (j) Representative flow cytometric analysis of red cells in bone marrow. (k–n) Percentages of reticulocytes (k), polychromatic and acidophilic normoblasts (l), basophilic and polychromatic normoblasts (m), and erythrocytes (n) in bone marrow. *p < .05, **p < .01, ***p < .001 versus the control group

To obtain specific data about the change of subtype of bone marrow hematopoietic cells, we used FACS to detect the change of phenotype of bone marrow cells. We found that the percentage of LSK cells (Lin‐Sca‐1^+^ c‐kit^+^) increased (Figure 2a,b) after microgravity simulation, but the differences between the control and treated groups were not significant. Bone marrow colony‐forming units (CFU) were determined on Day 28. Hindlimb suspension slightly increased CFU compared with the control group (Figure 2c,d), consistent with the observed increase in LSK cells.

Ly6G is expressed predominantly on neutrophils but can also be expressed on differentiating premonocytes and other cell types (Ortega et al., 2009). Approximately 41.8 ± 4.7% of bone marrow cells from animals exposed to sµg were positive compared with 34.1 ± 4.4% (*p* < .01) for control mice (Figure 2f).

We analyzed the level of Ly6C expression among the total bone marrow population. Ly6C expression is related to differentiation and is indicative of cells at an intermediate stage within the myeloid lineage. Ly6C is absent in cells of erythroid lineage and completely lost by cells upon final maturation into macrophages (Ortega et al., 2009). Overall, 19.3 ± 1.7% of bone marrow cells from sµg‐exposed animals were positive compared with 15 ± 1.8% (*p* < .01) for control mice (Figure 2g).

The expression of F4/80 (expressed on macrophages) was higher in bone marrow cells from treated animals than control animals (7.5 ± 0.7% vs. 5.4 ± 0.3%, *p* < .01; Figure 2h).

Vacek, Michurina, Serova, Rotkovska, and Bartonickova's (1991) work showed that compared to cells cultured under normal gravity, the numbers of rat bone marrow cells, granulocytes, macrophages, and hematopoietic progenitor cells were significantly lowered when cultured onboard the Cosmos‐2044 Biological Experimental Satellite. But Davis et al.'s (1996) work demonstrated that microgravity cultures could accelerate maturation/differentiation toward the macrophage lineage.

The percentage of B220^+^ (expressed on the B lymphocyte) lymphocytes in our study decreased (Figure 2i) after sµg. The difference between the two groups achieved statistical significance (10.9 ± 2.6% vs. 20.7 ± 3.5%, *p* < .01). HU can alter the quantity, distribution, and lymphopoiesis of lymphoid cells in bone marrow (Lescale et al., 2015). Ichiki et al. (1996) observed that the numbers of lymphoid progenitor cells isolated from rats undergoing SF decreased. Other data suggest that mature B cells were significantly suppressed, accompanied by increased natural killer cells in rodents exposed to SF (Gridley et al., 2009).

Domaratskaya et al. (2002) verified that the number of RBCs also decreased. Using FACS, we elucidated that sµg decreased mature erythrocyte counts in bone marrow but not basophilic normoblasts, polychromatic normoblasts, acidophilic normoblasts, or reticulocytes (Figure 2j–n). This is the first report about the effect of sµg on the different developmental stage of bone marrow RBCs. Davis et al. (1996), Lo Celso et al. (2009), Plett, Abonour, Frankovitz, and Orschell's (2004) study showed that erythroid progenitor cell numbers and erythroid development reduced at microgravity/modeled microgravity. These reports combined our results could explain why humans subjected to periods of microgravity develop anemia.

In short, our study showed that sµg increased the percentages of macrophages, granular leukocytes, and monocytes and slightly increased LSK cells and colony‐forming units but decreased the numbers of mature erythrocytes and B lymphocytes in bone marrow. The above mentioned results have some incompatibilities with others’ reported results due to different animal models and detection methods. More experiments need to be conducted to verify the effects of sµg on bone marrow.

### 3.3 Phenotype of spleen and peripheral blood lymphocytes

**Figure 3:**
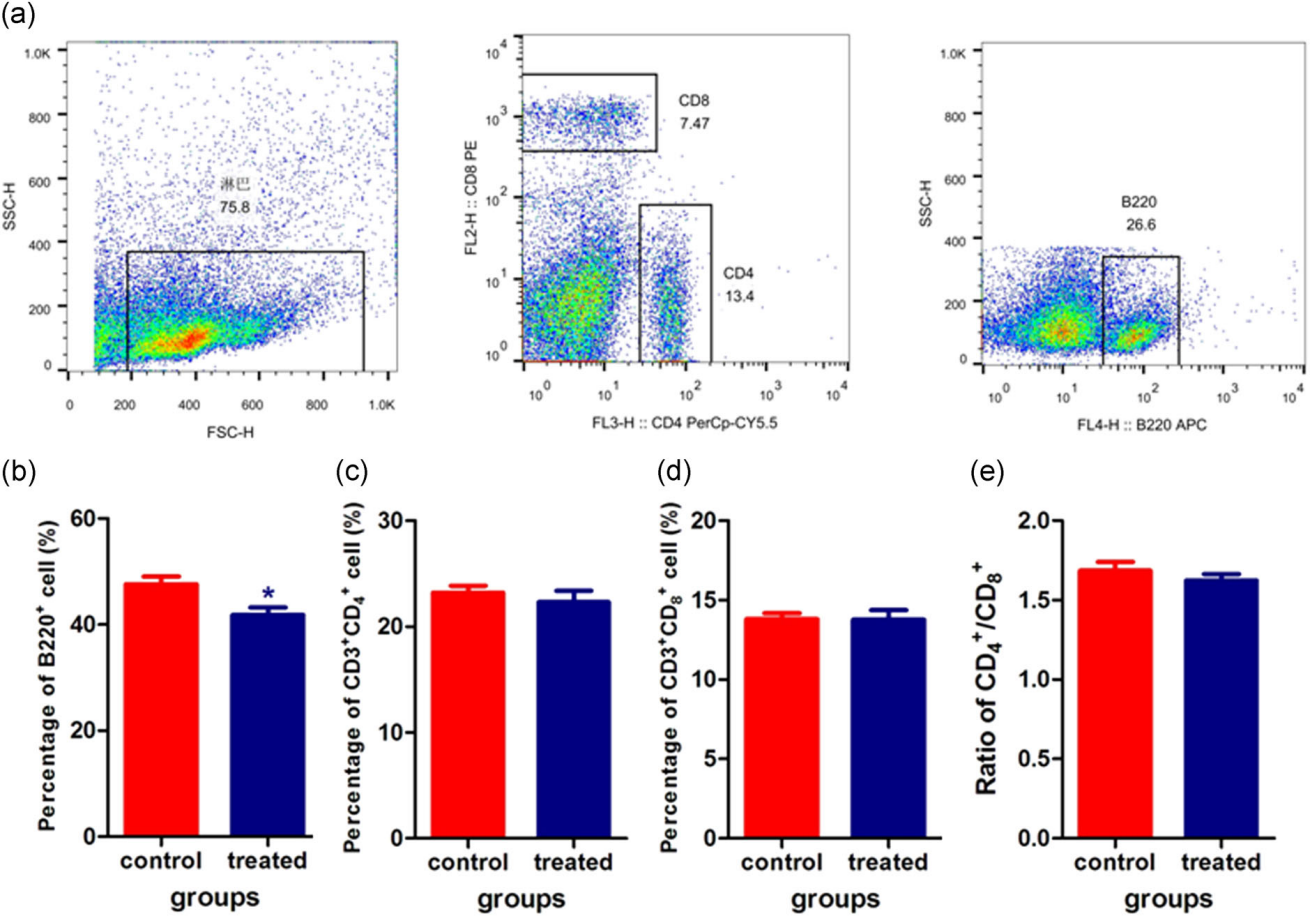
Phenotypes of spleen lymphocytes. Splenic lymphocytes were labeled with the fluorescent antibodies APC‐B220, PerCP‐Cy5.5‐CD4, and PE‐CD8a. Cell types were detected using fluorescence activated cell sorting (FACS). (a) Representative images of spleen lymphocytes. (b) The percentage of B220^+^ B cells. (c) The percentage of CD3^+^CD4^+^ T cells. (d) The percentage of CD3^+^CD8^+^ T cells. (e) The ratio of CD4^+^ T cells to CD8^+^ T cells. *p < .05 versus the control group

In this study, spleens were collected and lymphocytes were separated at Day 28 of microgravity simulation. Phenotypes were analyzed by FACS and the results showed that sµg decreased the percentage of B220^+^ lymphocytes (Figure 3b), which was in accordance with the results from bone marrow, but no effect was seen on CD4^+^ T lymphocytes, CD8^+^ T lymphocytes, or the CD4^+^/CD8^+^ ratio (Figure 3c–e).

**Figure 4:**
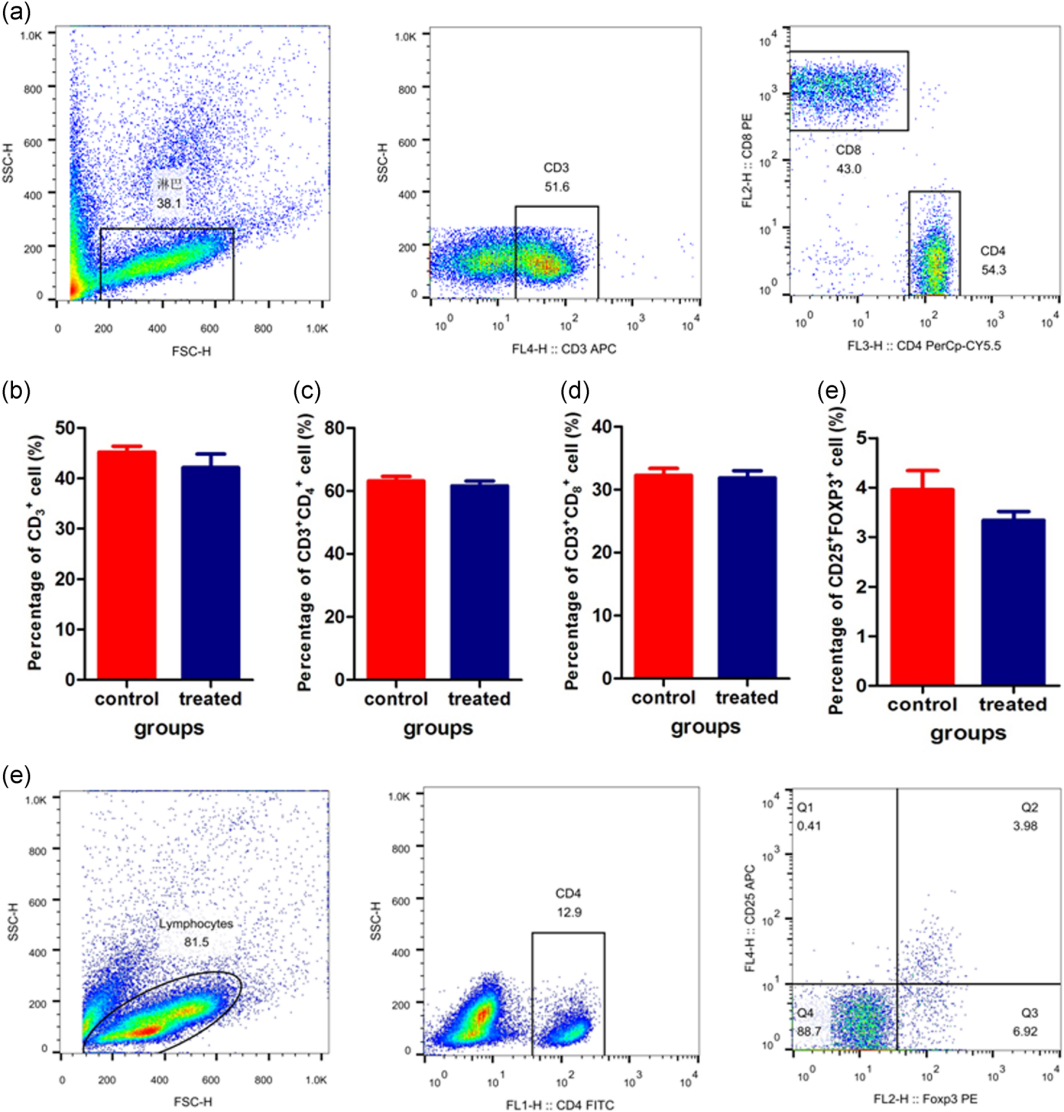
Phenotypes of peripheral blood T lymphocytes. On Day 28 of hindlimb unloading, heparin anticoagulant‐treated blood samples were labeled with APC‐CD3e, PerCP‐Cy5.5‐CD4, and PE‐CD8a. Immune phenotypes were analyzed by flow cytometry. (a) Representative images of peripheral blood T lymphocytes. (b) The percentage of CD3^+^ T cells. (c) The percentage of CD3^+^CD4^+^ T cells. (d) The percentage of CD3^+^CD8^+^ T cells. CD4^+^CD25^+^ Foxp3^+^ regulatory T cells (Tregs) were evaluated using a mouse Treg detection kit (e,f). Representative images are shown in (e)

Peripheral blood mononuclear cells were separated, and CD3, CD4, CD8 (Figure 4a–d) and Treg cells (Figure 2e,f) were detected by FACS. We observed that sµg had no effect on the phenotype of peripheral blood T lymphocytes.

Microgravity may affect the phenotypes of splenocytes and peripheral lymphocytes. The numbers of CD4, CD8, CD2, CD3, and B cells in the peripheral blood of rats on SF decreased compared to rats under normal gravity, but spleen lymphocytes did not differ (Ichiki et al., 1996). Other studies also showed that the number of T cells in peripheral blood was reduced during SF in both humans and rodents (Gridley et al., 2009; Ichiki et al., 1996). But in our study, populations of circulating T lymphocytes of treated mice have not significantly change compared with that of control mice (Figure 4). B lymphocytes decreased in the spleens of treated mice compared with control mice (Figure 3). These results are not coincidence with that of Gibson and Slater's, demonstrated that there are still exist some differences between real and sµg.

### 3.4 Effects of microgravity simulation on the cytoskeleton, differentiation‐related genes and hematopoietic growth factor expression in cultured BM‐MSCs

We used FACS to detect the phenotype of cultured BM‐MSCs and discovered that they expressed the phenotype of MSCs, CD29 and Sca‐1, and didn't express the phenotype of HSCs (CD34) and vascular endothelium (CD31; Figure S2).

**Figure 5:**
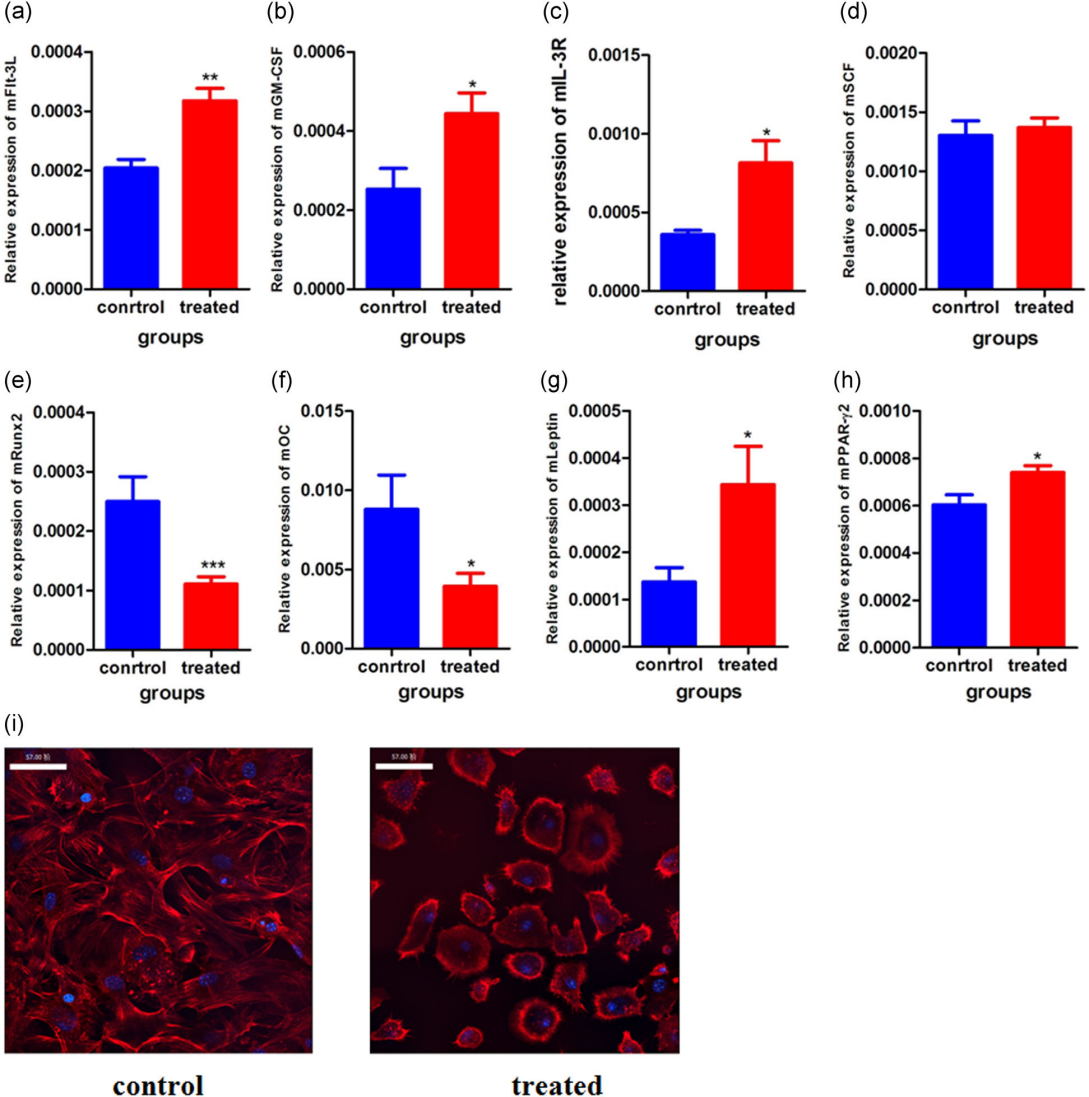
Effects of microgravity simulation on BM‐MSCs. Mice were sacrificed on Day 28 of microgravity simulation, and bone marrow was collected. BM‐MSCs were cultured, and β‐actin was used as an internal parameter. RT‐PCR was used to detect the expression levels of Flt‐3L (a), GM‐CSF (b), IL‐3R (c), SCF (d), RUNX2 (e), OC (f), leptin (g), and PPARγ2 (h) by RT‐PCR. (i) Confocal microscope analyses of microfilaments at 28 days after microgravity. Data are shown as the mean ± *SEM*. BM‐MSC, bone marrow‐derived mesenchymal stem cell; Flt‐3L, fms‐like tyrosine kinase‐3 ligand; GM‐CSF, granulocyte‐macrophage colony stimulating factor; IL‐3R, interleukin‐3R; OC, osteocalcin; PPARγ2, proliferator‐activated receptor γ type 2; RT‐PCR, real‐time polymerase chain reaction; RUNX2, RUNT‐related transcription factor 2; SCF, stem cell factor. *p < .05, **p < .01, ***p < .001 versus the control group

BM‐MSCs contribute to the important bone marrow microenvironment in which hematopoiesis takes place. Using real‐time PCR, we quantitated the expression of fms‐like tyrosine kinase‐3 ligand (Flt‐3L), granulocyte‐macrophage colony stimulating factor (GM‐CSF), interleukin‐3R (IL‐3R), and stem cell factor (SCF) in cultured BM‐MSCs. Under the influence of sµg, the expression levels of Flt‐3L (Figure 5a), GM‐CSF (Figure 5b), and IL‐3R (Figure 5c) were elevated, while SCF (Figure 5d) was unchanged compared with the control group. We hypothesized that morphological changes and expression changes in the hematopoietic promotion genes of BM‐MSCs may be partially responsible for the changes in hematopoietic cells. Further study is needed to verify this hypothesis.

Many experiments have verified that microgravity can change MSCs’ morphology and cytoskeletal structure. Isolated BM‐MSCs displayed a more flattened morphology after culture in microgravity simulated by prolonged clinorotation compared with cells cultured under normal gravity (C. Zhang, Li, Chen, & Wang, 2015). Other researchers have also observed MSCs morphology changes under microgravity (X. Zhang et al., 2013). Different groups have reached different conclusions regarding growth rate effects. Zhang et al. found the growth rate of BM‐MSCs to be decreased, but Ma's group observed that the growth rate of periodontal ligament stem cells increased. Microgravity can alter the biological characteristics of MSCs by inducing changes in microfilaments and in MSCs cell adhesion (C. Zhang et al., 2015).

Microfilaments in MSCs are highly sensitive to weightlessness. We found that in the control group, MSCs contained regular and well‐defined microfilaments under significant tension. Microfilament bundles were arranged in radial arrays along the long axis of the MSCs. After 28 days of sµg, microfilament expression was decreased, and the typical radial array along the long axis was disrupted (Figure 5i).

In addition to influencing MSCs morphology and cytoskeletal structure, gravity can also strongly influence MSCs differentiation. To investigate the possibility that sµg influences the differentiation pattern of BM‐MSCs, RT‐PCR was used to detect the expression of osteogenic fate‐related RUNT‐related transcription factor 2 (Runx2) and osteocalcin (OC) and adipogenic fate‐related Leptin and proliferator‐activated receptor γ type 2 (PPARγ2). The results showed that under sµg conditions, the expression levels of leptin (Figure 5g) and PPARγ2 (Figure 5h) were upregulated, and the levels of Runx2 (Figure 5e) and OC (Figure 5f) were decreased. Other groups also observed that hypergravity could induce MSCs to differentiate into force‐sensitive cells such as osteoblasts and myocardial cells, whereas microgravity caused MSCs to differentiate into force‐insensitive cells, such as adipocytes (Huang et al., 2009). hMSCs in a rotating wall vessel (RWV) bioreactor do not express osteogenic fate genes, such as alkaline phosphatase (ALP), collagen‐I (COL‐1), osteonectin (OS), and Runx2, or express them at lower levels. In contrast, the expression of lipogenic fate genes is upregulated under sµg conditions (Zayzafoon, Gathings, & McDonald, 2004; Zheng et al., 2007). These results showed that sµg can affect the differentiation ability of MSCs in vitro and in vivo, which are similar to that of our above mentioned findings.

The mechanism by which microgravity influences MSCs differentiation is thought to be related to the cytoskeleton, which is involved in intercellular signaling (Cau and Hall, 2005; Hosu, Mullen, Critser, & Forgacs, 2008). Studies have shown that microgravity can change MSCs differentiation potential by suppressing microfilament formation and RhoA activity or by increasing phosphorylation of p38 MAPK in these cells (Meyers, Zayzafoon, Douglas, & McDonald, 2005).

## 4 CONCLUSIONS

Simulating microgravity could influence the percentages of certain types of bone marrow cells, such as monocyte‐macrophages and B lymphocytes, and affect the morphology and gene expression of BM‐MSCs. The results of our study indicate that HU conditions can induce physiological changes in mice, such as gene expression changes in BM‐MSCs, thereby causing the cellular response of hematopoietic cells. These results may supply some favorable data for regenerative medicine and developmental biology.

## CONFLICT OF INTERESTS

The authors declare that there are no conflict of interests.

## Supporting information

Supporting information

Supporting information

Supporting information

## Data Availability

The data that support the findings of this study are available from the corresponding author upon reasonable request.
