# Supplementary figures and images for: Effect of simulated microgravity conditions of hindlimb unloading on mice hematopoietic and mesenchymal stromal cells
Source: Cell Biol Int. 2020 Aug 8;44(11):2243–52. doi: 10.1002/cbin.11432 (PMC7589432; doi:10.1002/cbin.11432)

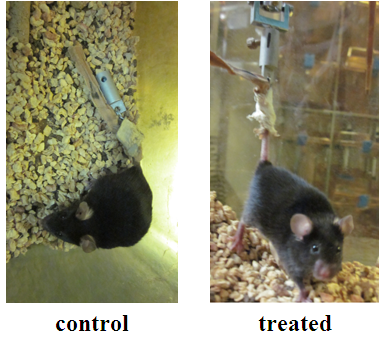

Supplement: Supplementary file 1 — Supporting information [file CBIN-44-2243-s001.TIF]

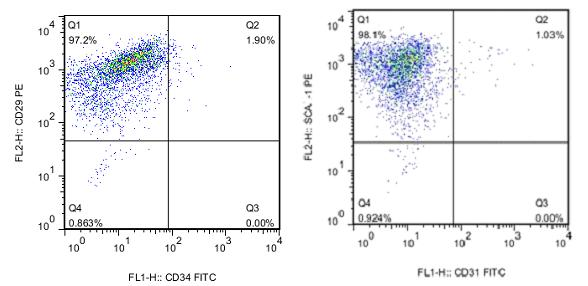

Supplement: Supplementary file 2 — Supporting information [file CBIN-44-2243-s002.TIF]
