# Supplementary material for: Effect of simulated microgravity conditions of hindlimb unloading on mice hematopoietic and mesenchymal stromal cells
Source: Cell Biol Int. 2020 Aug 8;44(11):2243–52. doi: 10.1002/cbin.11432 (PMC7589432; doi:10.1002/cbin.11432)
Supplement: Supplementary file 3 — Supporting information [file CBIN-44-2243-s003.docx]

**Supplementary figure1 Hindlimb unloading cage and the mice unloaded**

**Supplementary figure2 The phenotype of cultured BM-MSCs.**
